# Early Plasma Osmolality Levels and Clinical Outcomes in Children Admitted to the Pediatric Intensive Care Unit: A Single-Center Cohort Study

**DOI:** 10.3389/fped.2021.745204

**Published:** 2021-09-16

**Authors:** Huabin Wang, Zhongyuan He, Jiahong Li, Chao Lin, Huan Li, Ping Jin, Chun Chen

**Affiliations:** ^1^Division of Hematology/Oncology, Department of Pediatrics, The Seventh Affiliated Hospital of Sun Yat-sen University, Shenzhen, China; ^2^Department of Pediatric Intensive Care Unit, The Seventh Affiliated Hospital of Sun Yat-sen University, Shenzhen, China; ^3^Department of Orthopedics, The Seventh Affiliated Hospital of Sun Yat-sen University, Shenzhen, China; ^4^Center of Digestive Disease, The Seventh Affiliated Hospital of Sun Yat-sen University, Shenzhen, China; ^5^Shenzhen Baoan Women's and Children's Hospital, Jinan University, Shenzhen, China

**Keywords:** plasma osmolality, pediatric intensive care unit, mortality, prognostic value, cohort study

## Abstract

**Objective:** Identifying high-risk children with a poor prognosis in pediatric intensive care units (PICUs) is critical. The aim of this study was to assess the predictive value of early plasma osmolality levels in determining the clinical outcomes of children in PICUs.

**Methods:** We retrospectively assessed critically ill children in a pediatric intensive care database. The locally weighted-regression scatter-plot smoothing (LOWESS) method was used to explore the approximate relationship between plasma osmolality and in-hospital mortality. Linear spline functions and stepwise expansion models were applied in conjunction with a multivariate logistic regression to further analyze this relationship. A subgroup analysis by age and complications was performed.

**Results:** In total, 5,620 pediatric patients were included in this study. An approximately “U”-shaped relationship between plasma osmolality and mortality was detected using LOWESS. In the logistic regression model using a linear spline function, plasma osmolality ≥ 290 mmol/L was significantly associated with in-hospital mortality [odds ratio (OR) 1.020, 95% confidence interval (CI) 1.010–1.031], while plasma osmolality <290 mmol/L was not significantly associated with in-hospital mortality (OR 0.990, 95% CI 0.966–1.014). In the logistic regression model with plasma osmolality as a tri-categorical variable, only high osmolality was significantly associated with in-hospital mortality (OR 1.90, 95% CI 1.38–2.64), whereas low osmolality was not associated with in-hospital mortality (OR 1.28, 95% CI 0.84–1.94). The interactions between plasma osmolality and age or complications were not significant.

**Conclusion:** High osmolality, rather than low osmolality, can predict a poor prognosis in children in PICUs.

## Introduction

In recent years, although the mortality rate of critically ill children has shown a downward trend due to advancements in medical technology, the overall level is still quite high, especially among children aged under 5 years (1). Due to the complex and rapidly evolving condition of patients in pediatric intensive care units (PICUs), symptoms are often atypical, may change or progress at any time and can be life-threatening. The early identification of children whose condition may worsen or progress and providing timely interventions to these individuals are essential.

Plasma osmolality refers to the amount of molecules (mg) per kilogram of water and is clinically expressed in units of mOsm/(kg·H_2_O) or mmol/L. Measures of plasma osmolality, including crystal osmolality and colloid osmolality, which play important roles in regulating the distribution of water in blood vessels and inside and outside cells, are included in routine biochemical tests (2). Previous studies have shown that plasma osmolality is associated with the prognosis of patients with heart failure (3, 4), myocardial infarction (5, 6), and diabetic ketoacidosis (7), patients undergoing hemodialysis (8), elderly patients (9), and patients in the emergency room (10, 11).

Compared with adult patients, children have not fully developed, have poor body regulation and compensation abilities, have large individual differences, and have different requirements for water and electrolytes; therefore, children are more likely to have internal environment disorders (12, 13). Abnormal plasma osmolality is common among PICU patients. However, whether a plasma osmolality imbalance has an impact on the prognosis of critically ill children remains unclear. The aim of this study was to investigate the relationship between early plasma osmolality levels and the prognosis of PICU patients and provide clues for the early detection of high-risk pediatric patients to provide timely interventions.

## Materials and Methods

### Data Sources

**Figure 1:**
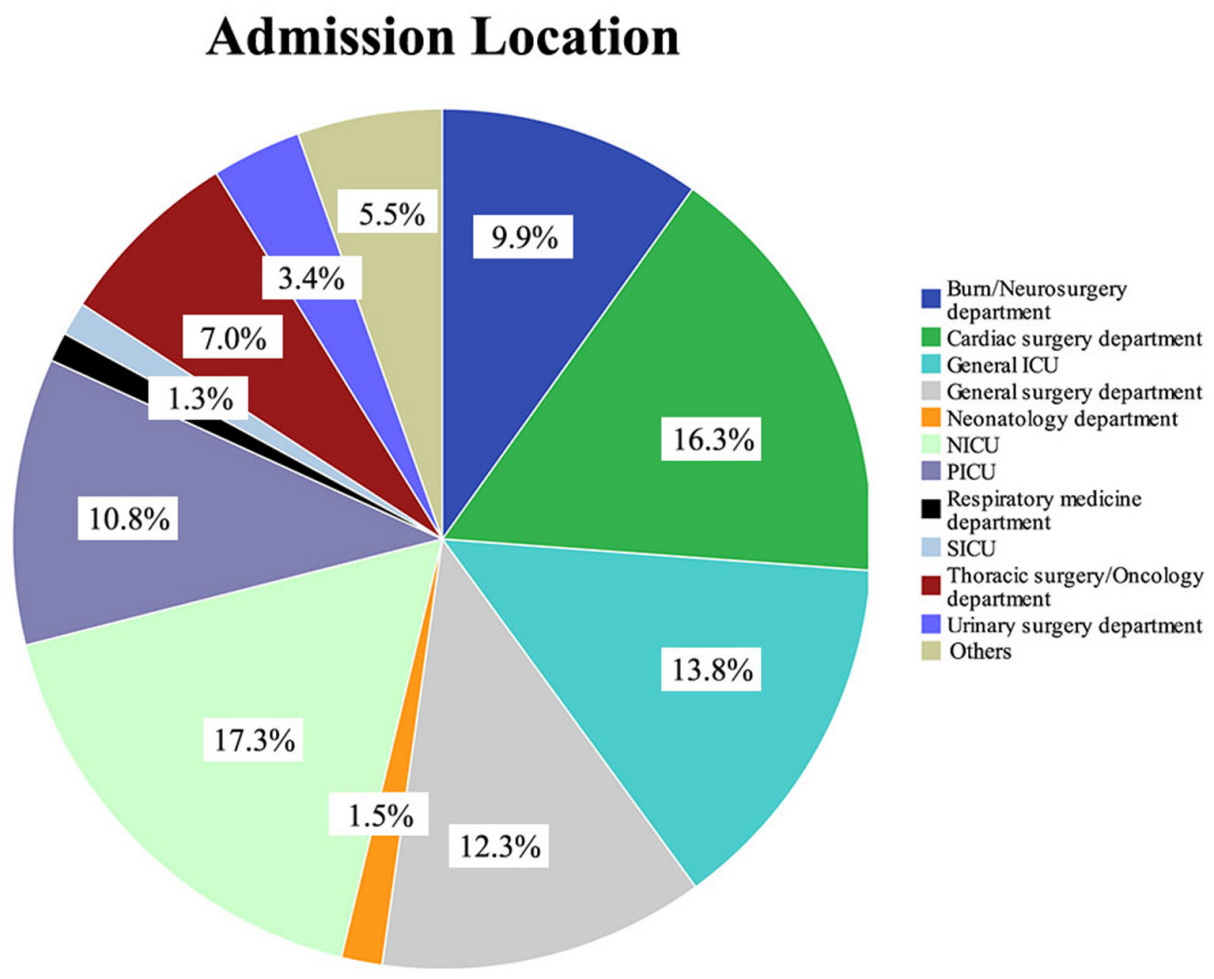
Type of department and proportion of patients in the pediatric intensive care database. NICU, neonatal intensive care unit; PICU, pediatric intensive care unit; SICU, surgery intensive care unit.

The pediatric intensive care (PIC) database is a large, open, dedicated, and single-center pediatric database (14) that collects the clinical data of all children admitted to multiple ICUs at the Children's Hospital of Zhejiang University School of Medicine, China; between 2010 and 2018, information concerning 13,499 admissions of a total of 12,881 different pediatric patients was collected (Figure 1). The collected data included the patients' demographics, diagnosis, laboratory test results, pathogenic microorganism test results, medications, clinical outcomes, and information regarding vital signs during operations. The PIC database was established on the basis of the success of the widely used Medical Information Mart for Intensive Care (MIMIC) database and can be downloaded free of charge after registration, application, and certification (http://pic.nbscn.org/). This project was approved by the Institutional Review Committee of the Children's Hospital of Zhejiang University School of Medicine. Because this project did not affect clinical treatment, the requirement for patient consent was waived. All protected health information was deidentified.

### Patient Population

**Figure 2:**
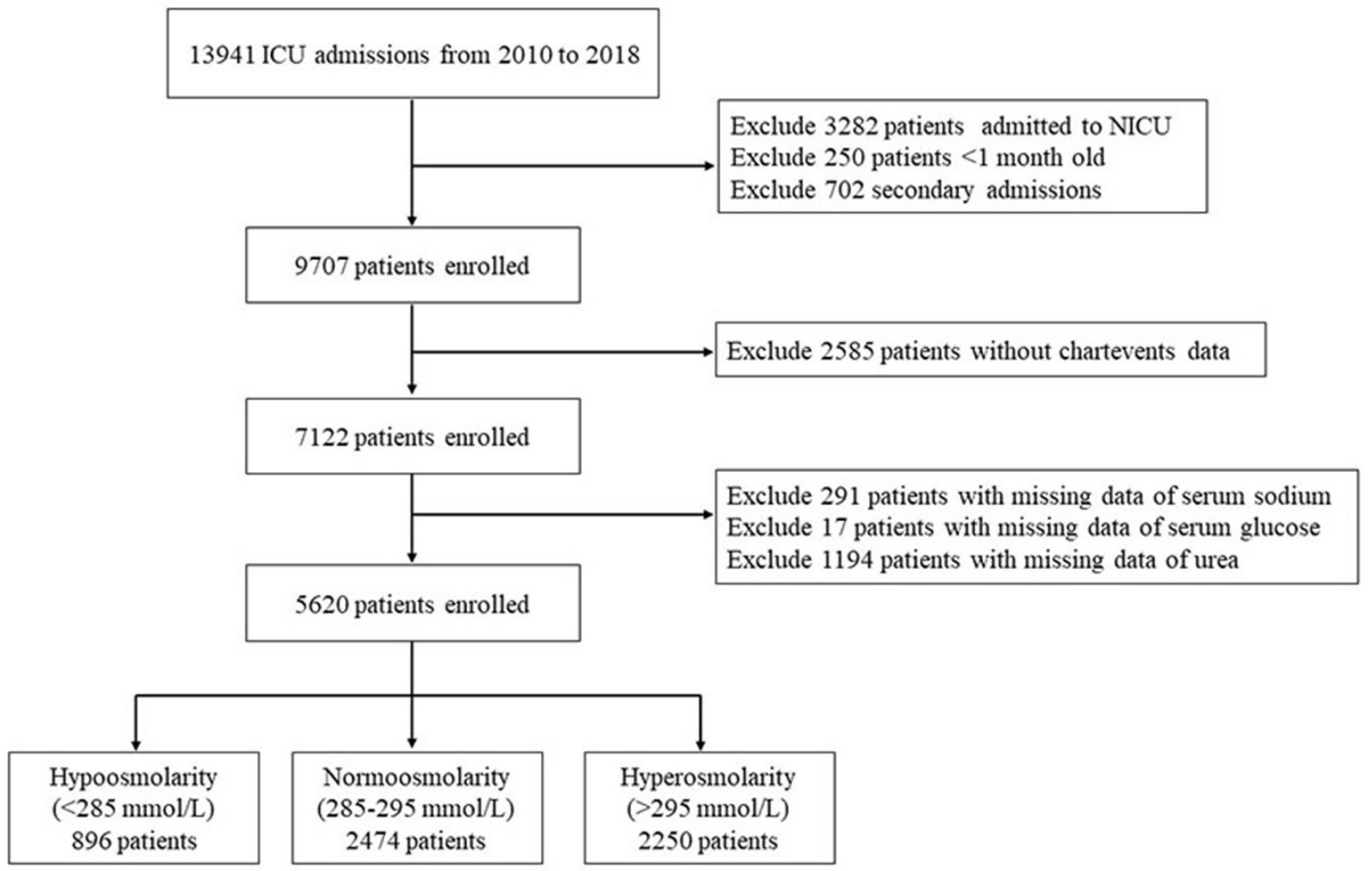
Flowchart of patients screened. NICU, neonatal intensive care unit.

This study was a retrospective cohort study. The inclusion criteria were as follows: (1) different types of ICU inpatients; (2) an age between 1 month and 18 years; and (3) the inclusion of data only from the first hospitalization if the patient was hospitalized multiple times. The exclusion criteria were as follows: (1) patients in the neonatal intensive care unit; (2) lack of chart data; and (3) patients without plasma osmolality data. The screening process is shown in Figure 2.

### Data Extraction and Addressing Missing Values

The extracted data included the patients' age, sex, type of ICU admission, laboratory test results (including peripheral white blood cell counts, platelet counts, serum sodium and potassium, chloride, glucose, alanine aminotransferase, albumin, creatinine, urea, activated partial thromboplastin time, arterial blood pH, bicarbonate, anion gap, and arterial blood lactate), and complications (including anemia, hypertension, sepsis, acute kidney injury, and malignancy). The diagnostic criteria for complications are provided in Supplementary Table 1. The formula used to calculate plasma osmolality was 2 (Na^+^ + K^+^) + glucose + urea, and the unit was mmol/L. All data used were the initial values after ICU admission. The primary outcome indicator was in-hospital mortality, which was defined as death occurring during hospitalization. The secondary outcome indicators included the 30-day mortality, length of ICU stay, and length of hospital stay.

Among the variables extracted in this study, the portion of missing values was <5%. Because none of these variables had a normal distribution, we used their median to replace the missing values.

### Grouping Method

**Figure 3:**
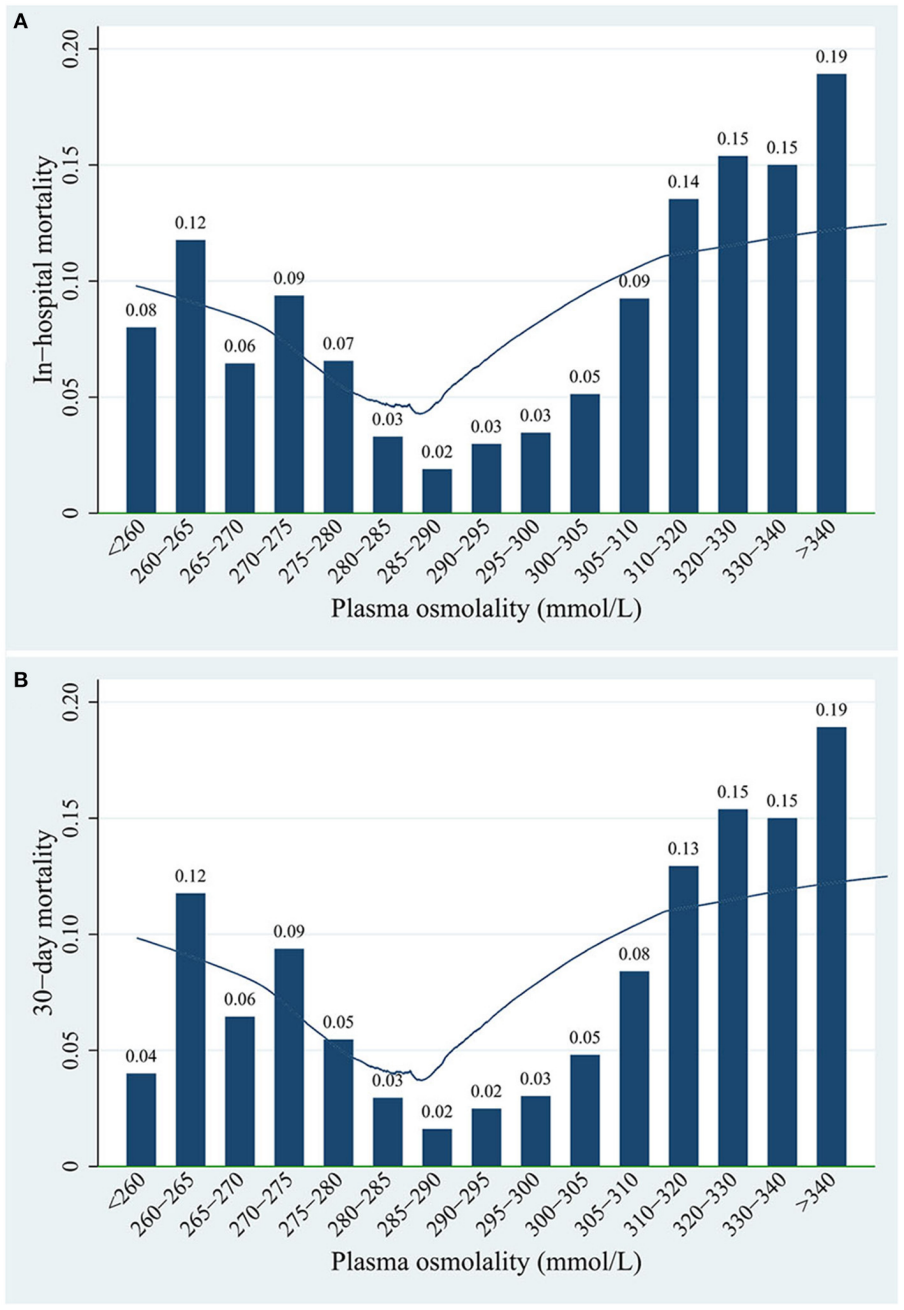
Association between plasma osmolality levels and **(A)** in-hospital mortality or **(B)** 30-day mortality of patients.

An approximately “U”-shaped relationship between plasma osmolality and in-hospital mortality or 30-day mortality was detected using the locally weighted-regression scatter-plot smoothing (LOWESS) method, with the lowest point of the curve at ~285–295 mmol/L (Figure 3). Therefore, we divided all patients based on their osmolality level into the following groups: low osmolality group (osmolality <285 mmol/L), normal osmolality group (285 mmol/L ≤ osmolality ≤ 295 mmol/L), and high osmolality group (osmolality >295 mmol/L).

### Statistical Analysis

The measurement data were first subjected to a normality test. Variables with a normal distribution are expressed as the mean ± standard deviation, and a *t*-test was used to compare two groups; variables without a normal distribution are expressed as the median (quartiles), and a non-parametric rank sum test was used to compare two groups. A chi-squared test was used to compare the count data. A backward stepwise method was used to screen for covariates to include in the multivariate logistic regression, and the normal range of plasma osmolality (285–295 mmol/L) was used as the reference group. Using LOWESS, a node for plasma osmolality was detected at ~290 mmol/L, and therefore, a linear spline function was initially used for the multivariate logistic regression. Covariate adjustment was performed using an extended model approach as follows: model 1 = plasma osmolality; model 2 = model 1 + (age, sex, and ICU type); model 3 = model 2 + (laboratory data); and model 4 = model 3 + (complications). A variance inflation factor ≥ 10 indicated that the regression model had severe multicollinearity.

Considering that the association between osmolality and mortality may differ with different comorbidities (15), we performed subgroup analyses by comorbidities. Since infants and adolescents have various physiological properties, another subgroup analysis was carried out by age. To verify the interaction between plasma osmolality and these variables, multiplicative interaction terms were included in the regression model. *P-*values < 0.05 were considered statistically significant. The statistical analysis was performed using STATA V.16, SPSS V.24, and R V.3.6.3.

## Results

### Baseline Characteristics

The data of 5,620 children were included in this analysis. Among all study subjects, the in-hospital mortality rate was 4.2%, and the 30-day mortality rate was 3.8%. Table 1 provides the baseline characteristics of the low osmolality group, normal osmolality group, and high osmolality group. The median age at the time of admission to the ICU was 18.6 months, and 54.2% of the patients were male. The patients in the normal osmolality group were compared with those in the low osmolality group and those in the high osmolality group. There were significant differences in age, sex, ICU type, all laboratory data, acute kidney injury and sepsis between the low or high osmolality groups and the normal osmolality group. The in-hospital mortality (4.8 vs. 2.5%) and 30-day mortality (4.2 vs. 2.1%) rates in the low osmolality group and the in-hospital mortality (5.9 vs. 2.5%) and 30-day mortality (5.4 vs. 2.1%) rates in the high osmolality group were higher than those in the normal osmolality group. Such a trend was also observed in the length of ICU stay.

**Table 1:** Baseline characteristics of the study population.

**Variable**	**Hypoosmolarity (*n* = 896)**	**Normoosmolarity (*n* = 2,474)**	**Hyperosmolarity (*n* = 2,250)**	*P*-value 1^*^	*P*-value 2^**^
	**(*n* = 896)**	**(*n* = 2,474)**	**(*n* = 2,250)**		
Age, months	14.7 (5.0–48.9)	17.3 (5.9–54.1)	21.7 (7.6–56.8)	0.013	<0.001
Male, *n* (%)	521 (58.1)	1373 (55.5)	1152 (51.2)	0.171	0.003
ICU type, *n* (%)				<0.001	<0.001
CIUC	233 (26.0)	942 (38.1)	1066 (47.4)		
PICU	366 (40.8)	620 (25.1)	665 (29.6)		
SICU	297 (33.2)	912 (36.8)	519 (23.1)		
**Laboratory data**					
WBC, 10^9^/L	9.0 (6.0–13.2)	9.3 (6.5–13.2)	10.0 (6.9–14.4)	0.098	<0.001
Platelet, 109/L	273 (184–367)	272 (190–361)	234 (155–339)	0.656	<0.001
Sodium, mmol/L	132 (130–133)	136 (135–137)	140 (139–142)	<0.001	<0.001
Chlorine, mmol/L	106 (102–110)	109 (106–112)	110 (107–113)	<0.001	<0.001
ALT, mg/dl	25.5 (14–44)	23 (14–34)	25 (15–36)	0.001	<0.001
Albumin, g/L	36.6 (31.9–39.4)	37.8 (34.3–40.6)	38.2 (35.7–41.5)	<0.001	<0.001
Creatinine, mg/dl	37 (28–46)	36 (28–44)	37 (28–47)	0.036	<0.001
Blood sugar, mmol/L	5.0 (1.6–8.1)	6.9 (5.8–8.9)	8.5 (6.3–11.2)	<0.001	<0.001
Arterial pH	7.40 (7.34–7.45)	7.38 (7.34–7.43)	7.36 (7.31–7.41)	<0.001	<0.001
Bicarbonate, mmol/L	21.9 (20.0–23.9)	22.2 (20.4–24.0)	21.9 (19.7–23.7)	0.046	<0.001
Lactate, mmol/L	1.6 (1.1–2.5)	1.6 (1.2–2.4)	2.0 (1.4–3.0)	0.106	<0.001
Anion gap, mmol/L	6.4 (5.4–8.2)	6.3 (3.6–9.2)	9.8 (6.6–13.3)	<0.001	<0.001
APTT, s	34 (29–44.7)	33.1 (28–40.1)	33.1 (28.1–41.9)	<0.001	0.139
**Comorbidities**, ***n*****(%)**					
Anemia	502 (56.0)	1373 (55.5)	1289 (57.3)	0.785	0.215
Hypertension	150 (16.7)	441 (17.8)	372 (16.5)	0.465	0.240
Acute kidney injury	147 (16.4)	327 (13.2)	512 (22.8)	0.330	<0.001
Sepsis	168 (18.8)	408 (16.5)	433 (19.2)	0.124	0.013
Malignancy	31 (3.5)	65 (2.6)	58 (2.6)	0.199	0.915
**Clinical outcome**					
ICU LOS (day)	3.0 (1.0–7.7)	1.7 (0.9–4.7)	2.6 (0.9–5.9)	<0.001	<0.001
Hospital LOS (day)	13.3 (7.4–20.0)	12.1 (7.6–18.1)	12.1 (7.6–18.8)	0.099	0.536
30 days mortality, *n* (%)	38 (4.2)	52 (2.1)	122 (5.4)	0.001	<0.001
Hospital mortality, *n* (%)	43 (4.8)	62 (2.5)	132 (5.9)	0.001	<0.001

* *P-value 1 represents the P-value of comparison between the group of hypoosmolarity and the group of normoosmolarity*.

** *P-value 2 represents the P-value of comparison between the group of hyperosmolarity and the group of normoosmolarity. ALT, Alanine aminotransferase; APTT, activated partial thromboplastin time; CICU, cardiac intensive care unit; LOS, length of stay; PICU, pediatric intensive care unit; SICU, surgery intensive care unit; WBC white blood cell*.

### Further Relationship Between Plasma Osmolality and Mortality

An approximately “U”-shaped relationship between plasma osmolality and mortality was detected using LOWESS (Figure 3). The in-hospital mortality and 30-day mortality rates were 2.0–3.0% when plasma osmolality was in the range of 285–295 mmol/L. However, both the in-hospital mortality and 30-day mortality rates reached 19.0% when the plasma osmolality was >340 mmol/L.

As shown in Table 2, plasma osmolality was first included in the multivariate logistic regression model as a continuous variable, and a linear spline function was used. When plasma osmolality was <290 mmol/L, the odds ratio (OR) of in-hospital mortality was not significant regardless of the covariate adjustment [crude OR 0.982, 95% confidence interval (CI) 0.960–1.004; adjusted OR 0.990, 95% CI 0.966–1.014]. When plasma osmolality was ≥ 290 mmol/L, the OR of in-hospital mortality remained significant after all covariate adjustments (crude OR 1.037, 95% CI 1.027–1.046; adjusted OR 1.020, 95% CI 1.010–1.031).

**Table 2:** Logistic regressions of plasma osmotic pressure for in-hospital mortality using linear spline function.

	**Crude OR**	**95% CI**	***P***	**Adjusted OR**	**95% CI**	***P***
Osmotic pressure (≤ 290, mmol/L)	0.982	0.960–1.004	0.110	0.990	0.966–1.014	0.431
Osmotic pressure (>290, mmol/L)	1.037	1.027–1.046	<0.001	1.020	1.010–1.031	<0.001
**Age**		–				
<12 months		Ref.–		Ref.		
≥12 months and <60 months	0.71	0.53–0.96	0.023	0.72	0.52–0.99	0.043
≥60 months and <120 months	0.72	0.48–1.09	0.117	0.63	0.40–0.98	0.042
≥120 months	0.74	0.45–1.23	0.246	0.63	0.36–1.08	0.093
Sex (female)	0.73	0.56–0.95	0.020	0.72	0.55–0.96	0.023
**ICU type**						
CICU		Ref.		Ref.		
PICU	5.66	3.97–8.07	<0.001	4.49	3.06–6.58	<0.001
SICU	1.40	0.91–2.17	0.126	1.59	1.00–2.54	0.050
WBC (<4 or >10, 10^9^/L)	1.62	1.24–2.13	<0.001	1.30	0.98–1.74	0.072
Platelet (<100, 10^9^/L)	2.03	1.39–2.95	<0.001	1.01	0.67–1.54	0.946
ALT (>80, U/L)	5.12	3.86–6.80	<0.001	2.10	1.52–2.91	<0.001
Albumin (<35, g/L)	1.64	1.26–2.15	<0.001	1.11	0.82–1.51	0.494
Creatinine (>88, μmol/L)	4.12	2.55–6.68	<0.001	0.87	0.49–1.54	0.631
Arterial pH (<7.35 or >7.45)	1.76	1.35–2.31	<0.001	1.09	0.81–1.46	0.561
Bicarbonate (<22 or >27, mmol/L)	2.35	1.76–3.15	<0.001	1.43	1.04–1.96	0.026
Lactate (≥2, mmol/L)	3.53	2.66–4.70	<0.001	2.40	1.77–3.26	<0.001
Anion gap (<8 or >16, mmol/L)	0.92	0.71–1.20	0.548	0.98	0.73–1.30	0.879
APTT (>45, s)	2.52	1.92–3.30	<0.001	1.88	1.38–2.55	<0.001
Anemia	0.83	0.64–1.08	0.163	0.76	0.57–1.01	0.055
Hypertension	0.67	0.47–0.94	0.021	0.77	0.53–1.11	0.155
Acute kidney injury	2.28	1.72–3.02	<0.001	1.59	1.17–2.16	0.003
Sepsis	0.93	0.65–1.31	0.659	1.24	0.85–1.82	0.268
Malignancy	1.43	0.72–2.83	0.311	1.58	0.77–3.25	0.215

*ALT, Alanine aminotransferase; APTT, activated partial thromboplastin time; CI, confidence interval; CICU, cardiac intensive care unit; LOS, length of stay; OR, odds ratio; PICU, pediatric intensive care unit; SICU, surgery intensive care unit; WBC white blood cell*.

In the extended multiple logistic regression model, with the normal osmolality group as the reference, the plasma osmolality level exhibited by the high osmolality group was significantly associated with increased in-hospital mortality (Table 3) as follows: model 1 (OR 2.43; 95% CI 1.78–3.30), model 2 (OR 2.45; 95% CI 1.79–3.36), model 3 (OR 1.91; 95% CI 1.39–2.64), and model 4 (OR 1.90; 95% CI 1.38–2.64). The relationship between the plasma osmolality level and 30-day mortality rate in the high osmolality group was similar. However, the association between osmolality and mortality in the low osmolality group was non-significant after the covariate adjustment. The results of the remaining covariates in model 4 are provided in Supplementary Table 2.

**Table 3:** OR (95% CI) for all-cause mortality across three serum osmolality levels.

	**Normoosmolarity**		**Hypoosmolarity**		**Hyperosmolarity**	
	**OR (95% CI)**	***P***	**OR (95% CI)**	***P***	**OR (95% CI)**	***P***
**Hospital mortality**						
Model 1	Reference	–	1.96 (1.32–2.92)	0.001	2.43 (1.78–3.30)	<0.001
Model 2	Reference	–	1.50 (1.00–2.24)	0.049	2.45 (1.79–3.36)	<0.001
Model 3	Reference	–	1.27 (0.84–1.93)	0.256	1.91 (1.39–2.64)	<0.001
Model 4	Reference	–	1.28 (0.84–1.94)	0.249	1.90 (1.38–2.64)	<0.001
**30–day mortality**						
Model 1	Reference	–	2.06 (1.35–3.16)	0.001	2.67 (1.92–3.71)	<0.001
Model 2	Reference	–	1.55 (1.01–2.39)	0.047	2.68 (1.91–3.75)	<0.001
Model 3	Reference	–	1.28 (0.82–2.00)	0.281	2.04 (1.44–2.88)	<0.001
Model 4	Reference	–	1.29 (0.82–2.02)	0.270	2.00 (1.41–2.83)	<0.001

*CI, confidence interval; OR, odds ratio*.

### Subgroup Analysis

**Figure 4:**
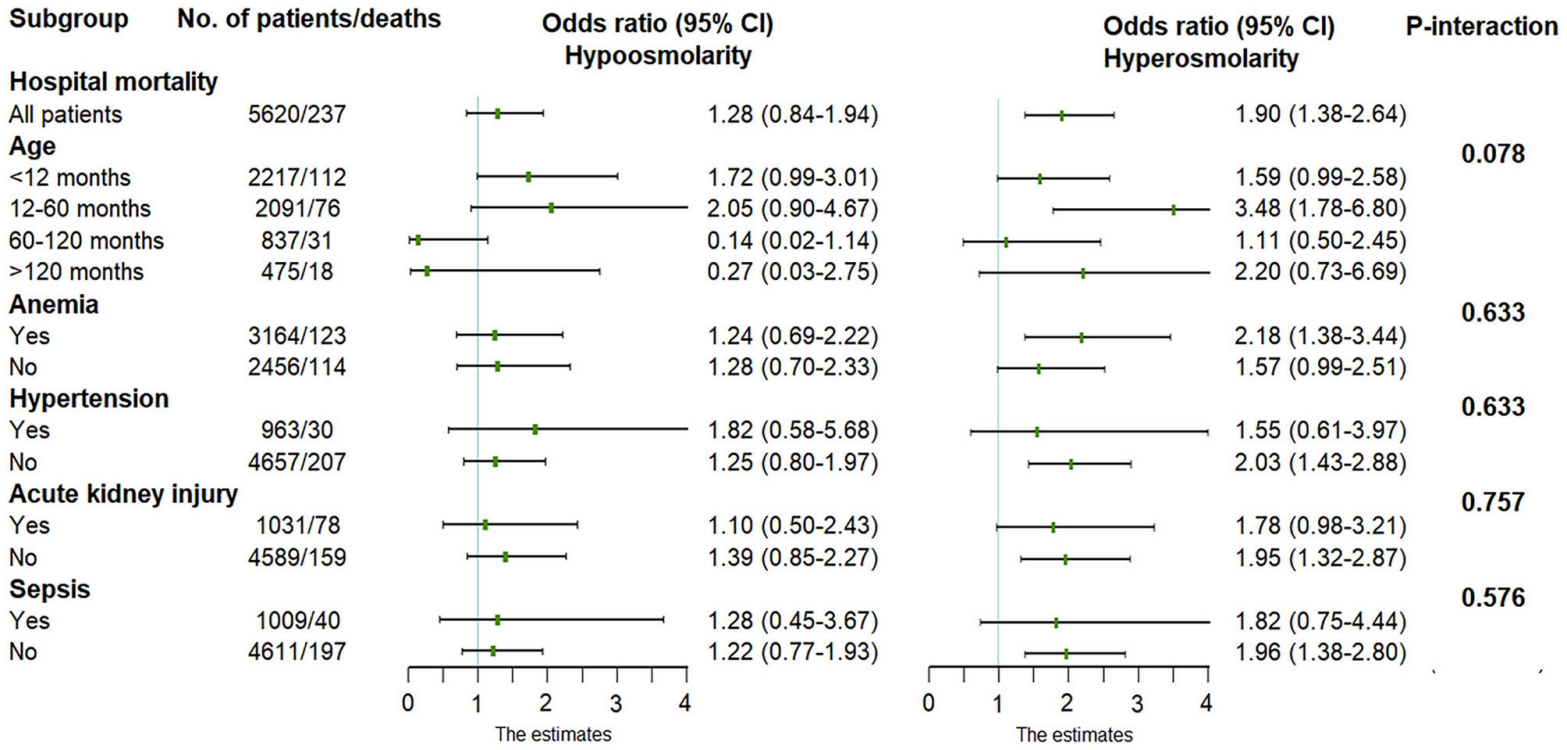
Adjusted odds ratio in the multivariate logistic regression. Normal osmolality was used as the reference level.

A multiplicative interaction term was introduced into the regression model to verify the interaction between plasma osmolality and age, anemia, hypertension, acute kidney injury, or sepsis. However, there was no interaction between age (or comorbidities) and plasma osmolality (*P-interaction* > 0.05), suggesting that the conclusions are stable and reliable across different ages and these comorbidities (Figure 4).

## Discussion

The primary objective of this study was to assess the predictive value of early plasma osmolality levels in determining the clinical outcomes of patients in PICUs. Our results indicate that the in-hospital mortality and 30-day mortality rates in both the high and low plasma osmolality groups were higher than those in the normal osmolality group. Plasma osmolality and mortality showed an ~“U”-shaped relationship. However, after all covariate adjustments, only the OR of in-hospital mortality in the high osmolality group remained significant, while the OR of that in the low osmolality group was non-significant.

Plasma osmolality plays an important role in regulating the distribution of intracellular and extracellular water (16). The movement of water between various body fluids and between the inside and outside of cells and abnormal changes in the content of electrolytes, particularly serum sodium, can lead to osmolarity imbalances and disturbances in the functional activities of the body (2, 17, 18). Increased osmolality indicates a decrease in water or an increase in solutes in the body, which is often observed in diabetic hyperosmolar coma, diabetes insipidus, heat stroke, high fever, and hyperosmolar dehydration. Decreased osmolality indicates an increase in water or a decrease in solutes in the body, which is often observed in heart failure, hypoproteinemia, hyponatremia, the oliguric phase of renal failure, and hypotonic dehydration. An imbalance in plasma osmolality is very common in critically ill patients (19–21). In this study, more than half (55.98%) of the patients showed abnormal osmolality. Among these patients, 896 patients (15.94%) had low osmolality, and 2,250 patients (40.04%) had high osmolality. The number of patients with high osmolality was 2.5 times the number of patients with low osmolality.

Our study shows that high plasma osmolality is significantly associated with increased in-hospital mortality among pediatric patients in PICUs, which is consistent with some previous research results. Ahmet et al. investigated 245 patients with acute pulmonary embolism (22). After all covariate adjustments, compared with low and normal osmolality, high osmolality was an independent risk factor for in-hospital mortality (OR 3.6, 95% CI 1.3–18.8). In addition, the high osmolality group had a higher incidence of cardiac arrest, hypotension, and cardiogenic shock. In a 1-year follow-up study of patients with heart failure, the investigators equally divided the patients into four groups based on osmolality quartiles, and the mortality rates were 18% (Q1), 18% (Q2), 23% (Q3), and 28% (Q4) (20). In a multivariate Cox regression model, high osmolality was significantly associated with all-cause mortality. Plasma hyperosmolarity leads to intracellular fluid depletion, which seriously affects intracellular metabolism and physiological functions (23), such as the metabolic activities of the heart (24), kidneys (9), and lungs (25, 26). Regarding brain cell function, contracted neurons stretch, causing membrane potential changes that could result in neurological dysfunction (27), cerebral edema, intracerebral hemorrhage, thrombosis, and brain injuries that are usually fatal (28). Children's organs are developmentally immature; therefore, the impact is even more severe.

The relationship between low plasma osmolality and patient mortality has rarely been studied. Price et al. conducted a retrospective study of 141 children hospitalized for acute decompensated heart failure (21). Hyponatremia upon admission was associated with a higher likelihood of death, heart transplantation, or the need for mechanical circulatory support (OR 3.1, 95% 1.2–8.3). In total, 1,240 hemodialysis patients were included in the Japanese Dialysis Outcomes and Practice Patterns Study (8). Low osmolality was associated with higher mortality before hemodialysis (for every 10 mOsm/L decrease in osmotic pressure, hazard risk 1.52, 95% CI 1.30–1.78). However, our results indicate that low osmolality is not an independent risk factor for death in critically ill children. The following reasons may account for the contradictory conclusions. First, in patients with heart failure, low osmolality is strongly associated with an increased fluid load, which can further aggravate the symptoms of heart failure and thus cause adverse outcomes. Our study included patients in the PICU, causes of low osmolality were diverse and may not affect vital organ function. Second, low osmolality may have different impacts on different types of diseases. Although the effects of some complications were corrected in our study, certain disease information could not be obtained.

To the best of our knowledge, this study is the largest study to date to investigate the relationship between plasma osmolality and the prognosis of patients in PICUs. However, our work has some limitations. First, this study is a single-center retrospective cohort clinical study; thus, the representativeness of the study cohort may be an issue, and the exact mechanism by which high osmolality contributes to high in-hospital mortality could not be determined. Given the results of this clinical study, we are highly interested in carrying out relevant cellular and animal experiments in the future to further explore the mechanisms by which high osmolality affects prognosis. Second, regarding plasma osmolality, we selected the initial value upon admission to the PICU; therefore, the relationship between the dynamic changes in plasma osmolality and prognosis is still unknown. Third, our study subjects were all patients in PICUs who had various types of diseases; therefore, further studies are needed to elucidate the prognostic value of plasma osmolality for specific types of diseases in PICU patients. Fourth, due to the limitations of public databases, we could not accurately obtain data, including pupillary reflex, mental status, eye open response, verbal response, and motor response; thus, PRISM III, GCS, PELOD-2 and other critical scores could not be derived. In this study, we listed some laboratory indicators, such as lactate, white blood cells, activated partial thromboplastin clotting time, and creatinine, while some comorbidities, such as acute kidney injury and sepsis, also reflected the severity of the disease to some extent.

## Conclusions

In this study, we found that plasma hyperosmolality was an independent risk factor for in-hospital mortality in critically ill children in PICUs, suggesting that plasma osmolality may be a useful prognostic biomarker among patients in PICUs. Additionally, the conclusion of this study provides a potential therapeutic target for critically ill children, i.e., correcting abnormal plasma osmolality may improve the prognosis of patients in PICUs; however, this proposal requires further verification through future studies.

## Data Availability Statement

The data analyzed in this study is subject to the following licenses/restrictions: Reanalysis of the data requires approval by the PIC database. Requests to access these datasets should be directed to the first author wanghb53@mail2.sysu.edu.cn.

## Ethics Statement

The studies involving human participants were reviewed and approved by the Institutional Review Board of the Children's Hospital of Zhejiang University School of Medicine. Written informed consent from the participants' legal guardian/next of kin was not required to participate in this study in accordance with the national legislation and the institutional requirements.

## Author Contributions

PJ and CC conceptualized and designed the study and reviewed and revised the manuscript. HW coordinated and supervised data collection, drafted the initial manuscript, and reviewed and revised the manuscript. ZH carried out the analyses and interpreted the results and drafted the initial manuscript. JL, CL, and HL contributed to data collection and reviewed and revised the manuscript. All authors contributed to the article and approved the submitted version.

## Funding

This research was supported by the Sanming Project of Medicine in Shenzhen (SZSM202011004), the Natural Science Foundation of Guangdong Province (2020A1515010151), and Shenzhen Healthcare Research Project (SZLY2018001).

## Conflict of Interest

The authors declare that the research was conducted in the absence of any commercial or financial relationships that could be construed as a potential conflict of interest.

## Publisher's Note

All claims expressed in this article are solely those of the authors and do not necessarily represent those of their affiliated organizations, or those of the publisher, the editors and the reviewers. Any product that may be evaluated in this article, or claim that may be made by its manufacturer, is not guaranteed or endorsed by the publisher.
