# Supplementary material for: Early Plasma Osmolality Levels and Clinical Outcomes in Children Admitted to the Pediatric Intensive Care Unit: A Single-Center Cohort Study
Source: Front Pediatr. 2021 Sep 16;9:745204. doi: 10.3389/fped.2021.745204 (PMC8481873; doi:10.3389/fped.2021.745204)
Supplement: Supplementary file 1 [file Data_Sheet_1.pdf]

Supplementary Material

Supplemental Table 1. Diagnostic criteria for complications.

Complications	Diagnostic criteria
Acute kidney injury	We used the pROCK criterion, which defines acute kidney injury as an increase in creatinine levels of ≥ 20 μmol/L and ≥ 30% within 7 days.
Anemia	1-4 months < 90 g/L;<br>4-6 months < 100 g/L;<br>6-60minths < 110 g/L;<br>60-144months < 115 g/L;<br>> 144 months < 120 g/L.
Hypertension	Systolic pressure > 120 mmHg; diastolic pressure > 80 mmHg.
Sepsis	children with suspected or confirmed infections within 48 hours of PICU admission presenting with systemic inflammatory response syndrome.

Supplemental Table 2.1. The multivariate logistic regression model of model 4 associated with hospital mortality using a stepwise backward elimination method.

Variables	Odds ratio (95% CI)	*P*-value
**Age**		
<12 months	Ref.	
≥12 months and <60 months	0.72 (0.52-0.99)	0.043
≥60 months and <120 months	0.62 (0.40-0.97)	0.035
≥120 months	0.64 (0.37-1.11)	0.112
**Sex (female)**	0.71 (0.54-0.94)	0.018
**ICU type**		
CICU	Ref.	
PICU	4.65 (3.16-6.83)	<0.001
SICU	1.61 (1.01-2.57)	0.044
**WBC (<4 or >10, 10^9/L)**	1.30 (0.98-1.74)	0.073
**Platelet (<100, 10^9/L)**	1.01 (0.67-1.54)	0.957
**ALT (>80, U/L)**	2.23 (1.62-3.08)	<0.001
**Albumin (<35, g/L)**	1.19 (0.88-1.62)	0.268
**Creatinine (>88, µmol/L)**	1.05 (0.61-1.82)	0.853
**Arterial PH (<7.35 or >7.45)**	1.08 (0.81-1.45)	0.598
**Bicarbonate (<22 or >27, mmol/L)**	1.46 (1.06-1.99)	0.019
**Lactate (≥2, mmol/L)**	2.34 (1.72-3.18)	<0.001
**Anion gap (<8 or >16, mmol/L)**	1.04 (0.78-1.38)	0.802
**APTT (>45, s)**	1.84 (1.35-2.51)	<0.001
**Anemia**	0.76 (0.57-1.01)	0.062
**Hypertension**	0.76 (0.53-1.10)	0.148
**Acute kidney injury**	1.57 (1.16-2.13)	0.004
**Sepsis**	1.21 (0.82-1.77)	0.334

**Malignancy**	1.49 (0.73-3.07)	0.276
**Osmotic pressure**		
<285 mmol/L	1.28 (0.84-1.94)	0.249
285-295 mmol/L	Ref.	
>295 mmol/L	1.90 (1.38-2.64)	<0.001

ALT, Alanine aminotransferase; APTT, activated partial thromboplastin time; CI, confidence interval; CICU, cardiac intensive care unit; PICU, pediatric intensive care unit; SICU, surgery intensive care unit; WBC white blood cell.

Supplemental Table 2.2. The multivariate logistic regression model of model 4 associated with 30-day mortality using a stepwise backward elimination method.

Variables	Odds ratio (95% CI)	*P*-value
**Age**		
<12 months	Ref.	
≥12 months and <60 months	0.74 (0.52-1.04)	0.081
≥60 months and <120 months	0.59 (0.37-0.96)	0.032
≥120 months	0.77 (0.44-1.33)	0.343
**Sex (female)**	0.71 (0.52-0.95)	0.022
**ICU type**		
CICU	Ref.	
PICU	5.02 (3.30-7.62)	<0.001
SICU	1.57 (0.94-2.63)	0.083
**WBC (<4 or >10, 10^9/L)**	1.36 (1.00-1.85)	0.049
**Platelet (<100, 10^9/L)**	1.04 (0.68-1.61)	0.851
**ALT (>80, U/L)**	2.37 (1.70-3.31)	<0.001
**Albumin (<35, g/L)**	1.26 (0.91-1.73)	0.162
**Creatinine (>88, µmol/L)**	1.18 (0.68-2.03)	0.561
**Arterial PH (<7.35 or >7.45)**	1.06 (0.78-1.45)	0.711
**Bicarbonate (<22 or >27, mmol/L)**	1.55 (1.11-2.17)	0.010
**Lactate (≥2, mmol/L)**	2.53 (1.82-3.52)	<0.001
**Anion gap (<8 or >16, mmol/L)**	1.03 (0.76-1.40)	0.847
**APTT (>45, s)**	1.89 (1.38-2.59)	<0.001
**Anemia**	0.85 (0.63-1.16)	0.314
**Hypertension**	0.77 (0.53-1.13)	0.185
**Acute kidney injury**	1.70 (1.24-2.34)	0.001
**Sepsis**	1.30 (0.89-1.89)	0.177
**Malignancy**	1.21 (0.54-2.72)	0.644

**Osmotic pressure**		
<285 mmol/L	1.29 (0.82-2.02)	0.270
285-295 mmol/L	Ref.	
>295 mmol/L	2.00 (1.41-2.83)	<0.001

ALT, Alanine aminotransferase; APTT, activated partial thromboplastin time; CI, confidence interval; CICU, cardiac intensive care unit; PICU, pediatric intensive care unit; SICU, surgery intensive care unit; WBC white blood cell.
